# Identification of a seven-cell cycle signature predicting overall survival for gastric cancer

**DOI:** 10.18632/aging.204060

**Published:** 2022-05-10

**Authors:** Lian-Qun Zhang, Sheng-Li Zhou, Jun-Kuo Li, Pei-Nan Chen, Xue-Ke Zhao, Li-Dong Wang, Xiu-Ling Li, Fu-You Zhou

**Affiliations:** 1 Department of Gastroenterology, Henan Provincial People’s Hospital, People’s Hospital of Zhengzhou University, School of Clinical Medicine, Henan University, Zhengzhou 450003, Henan, China; 2 Department of Pathology, Henan Provincial People’s Hospital, People’s Hospital of Zhengzhou University, School of Clinical Medicine, Henan University, Zhengzhou 450003, Henan, China; 3 Department of Thoracic Surgery, Anyang Tumor Hospital, Anyang 455000, Henan, China; 4 Department of Thoracic Surgery, The Affiliated Cancer Hospital of Zhengzhou University, Henan Cancer Hospital, Zhengzhou 450008, Henan, China; 5 State Key Laboratory of Esophageal Cancer Prevention and Treatment and Henan Key Laboratory for Esophageal Cancer Research of the First Affiliated Hospital, Zhengzhou University, Zhengzhou 450003, Henan, China

**Keywords:** gastric cancer, cell cycle, prognostic model, TCGA, GEO

## Abstract

While genetic alterations in several regulators of the cell cycle have a significant impact on the gastric carcinogenesis process, the prognostic role of them remains to be further elucidated. The TCGA-STAD training set were downloaded and the mRNA expression matrix of cell cycle genes was extracted and corrected for further analysis after taking the intersection with GSE84437 dataset. Differentially expressed mRNAs were identified between tumor and normal tissue samples in TCGA-STAD. Univariate Cox regression analysis and lasso Cox regression model established a novel seven-gene cell cycle signature (including GADD45B, TFDP1, CDC6, CDC25A, CDC7, SMC1A and MCM3) for GC prognosis prediction. Patients in the high-risk group shown significantly poorer survival than patients in the low-risk group. The signature was found to be an independent prognostic factor for GC survival. Nomogram including the signature shown some clinical net benefit for overall survival prediction. The signature was further validated in the GSE84437 dataset. In tissue microarray, CDC6 and MCM3 protein expression were significant differences by the immunohistochemistry-based H-score between tumor tissues and adjacent tissues, and CDC6 is an independent prognostic factor for GC. Interestingly, our GSEA revealed that low-risk patients were more related to cell cycle pathways and might benefit more from therapies targeting cell cycle. Our study identified a novel robust seven-gene cell cycle signature for GC prognosis prediction that may serve as a beneficial complement to clinicopathological staging. The signature might provide potential biomarkers for the application of cell cycle regulators to therapies and treatment response prediction.

## INTRODUCTION

The mammalian cell cycle is a highly structured and regulated process that ensures the duplication of the genetic material and cell division. Cell cycle regulation involves both mechanisms that govern growth regulation and genetic integrity. Cancer is characterized by abnormal cell cycle activity, possibly because of upstream signaling pathway gene mutations or mutations in the genes that encode cyclins. Genetic changes in several cell cycle regulators have a key influence on the pathogenetic process of gastric cancer (GC) [1]. Studies have shown that cell cycle regulators are potential GC prognosis biomarkers of great clinical value [2], and targeting cell cycle regulators in GC treatment has also increasingly attracted attention [3].

Among the five most common cancers worldwide, GC is the third major cause of cancer mortality [4]. Despite surgical and adjuvant therapies, the prognosis of GC patients remains poor, and patients’5-year overall survival rate is below 25% [5]. Until now, the prediction of prognosis has primarily depended on histopathologic diagnosis and neoplasm staging systems. However, many patients in the progressive stage who have similar clinicopathologic features show great differences in prognosis.

Although much effort has been spent developing optimum tools for the prediction of GC prognosis, no consensus has been reached as to the best method. In most of the existing literature, clinical baseline characteristics (e.g., tumor size, lymphonodus count, and lymphatic vascular space invasion) and unimolecular biomarkers (e.g., CD44 [6], PPAR γ [7], IL-13Rα2 [8], HDAC6 [9]) are utilized to construct prognostic models. Nevertheless, the predictive power of monogenic biomarkers is insufficient. Based on recent development singenomic sequencing, the integration of prognosis-related genetic markers with traditional parameters has huge potential for the prediction of GC prognosis [10, 11].

In this study, differentially expressed cell cycle genes were identified from tumors and normal tissue specimens from The Cancer Genome Atlas Stomach Adenocarcinoma (TCGA-STAD) dataset. Then, univariate Cox regression analysis and lasso regression analysis were conducted on survival data to identify cell cycle genes significantly correlated with the overall survival (OS) of GC patients. These genes were used to construct a prognostic model, which was further verified in the GSE84437 dataset from Gene Expression Omnibus (GEO). Additionally, tissue microarrays were used in Chinese patients with GC to explore the relationship between selected cell cycle genes and prognosis based on their protein expression levels.

## RESULTS

### Prognostic model building and validation based on cell cycle genes in TCGA-STAD

The expression levels of 55 cell cycle genes in 334 patients with OS longer than 30 days (Table 1) from TCGA-STAD were used to train a prognostic model. Based on a univariate Cox regression model, seven genes (i.e., *GADD45B, TFDP1, CDC6, CDC25A, CDC7, SMC1A and MCM3*) were correlated with survival. Lasso regression analysis, including *GADD45B, TFDP1, CDC6, CDC25A, CDC7, SMC1A and MCM3*, was conducted to construct the prognostic model. The resulting risk score is calculated by 0.0090×expression level of *GADD45B* −0.0116×expression level of *TFDP1* +0.0053×expression level of *CDC6* −0.0177×expression level of *CDC25A* −0.0127×expression level of *CDC7* −0.0157×expression level of *SMC1A* −0.0018×expression level of *MCM3*. All patients were classified into either the high-or low-risk groups on the basis of the optimum cutoff value of the risk score, which was set at −0.474.

**Table 1:** Clinical features n (%) of GC patients with OS longer than 30 days.

**Parameters**	**TCGA-STAD patients with mRNA expression data (n =334)**	**GSE84437 patients with mRNA expression data (n = 431)**	**Chinese GC patients with immunohistochemical data (n=232)**
Gender			
Male	216(64.67)	294(68.21)	161(69.40)
Female	118(35.33)	137(31.79)	71(30.60)
Age (mean ± s.d.)	65.17 ±10.22	60.02 ± 11.58	58.09±12.18
pTMN			
I	44(13.17)	-	32(13.79)
II	106(31.74)	-	80(34.48)
III	137(41.02)	-	98(42.24)
IV	33(9.88)	-	22(9.48)
unknow	14(4.19)	-	-
T_stage			
1	14(4.19)	11(2.55)	26(11.21)
2	72(21.56)	38(8.82)	27(11.64)
3	156(46.71)	92(21.35)	79(34.05)
4	88(26.35)	290(67.29)	100(43.10)
X	4(1.20)	-	
N_stage			
0	98(29.34)	80(18.56)	76(32.76)
1	90(26.95)	187(43.39)	49(21.12)
2	68(20.36)	132(30.63)	47(20.26)
3	67(20.06)	32(7.42)	60(25.86)
X	11(3.29)	-	-
M_stage			
0	300(89.82)	-	210(90.52)
1	22(6.59)	-	22(9.48)
X	12(3.59)	-	-
Grade			
G1	9(2.69)	-	2(0.86)
G2	117(35.03)	-	53(22.84)
G3	199(59.58)	-	177(76.29)
GX	9(2.69)		
Vessel carcinoma embolus			
Yes	-	-	30(12.93)
No	-	-	202(87.07)
Vital status			
Alive	220(65.87)	224(51.97)	137(59.05)
Dead	124(37.13)	207(48.03)	95(40.95)

GC, gastric cancer; mRNA, messenger RNA; OS, overall survival; TCGA, the Cancer Genome Atlas.

**Figure 1:**
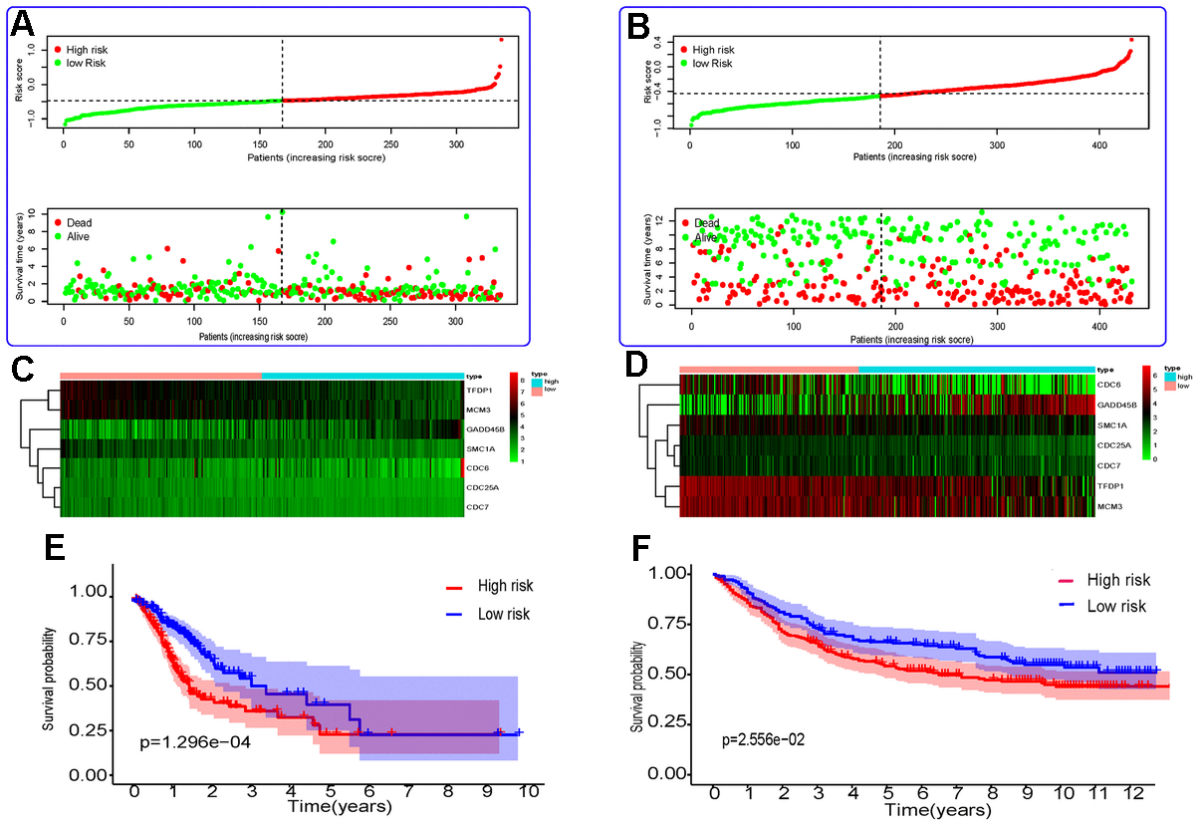
**Risk score analysis and Kaplan–Meier survival analysis for seven differentially expressed genes in GC.** (**A**, **B**) The distribution of risk score and patient’s survival time, as well as status for TCGA-STAD (**A**) and GSE84437 (**B**). (**C**, **D**) Heatmap of the autophagy-associated gene expression profiles in prognostic signature for TCGA-STAD (**C**) and GSE84437 (**D**). (**E**, **F**) Kaplan-Meier curves of seven differentially expressed genes from TCGA-STAD (**E**) and GSE84437 (**F**).

Then, 431 patients with OS longer than 30 days (Table 1) were selected from the GSE84437 dataset to validate the model. These patients were also divided into high- and low-risk groups on the basis of the optimum cutoff value of 1.087. Next, the Kaplan–Meier method was used to determine the survival curves of the two groups. As can be seen in Figure 1, the survival curves of the high-risk patients are different from those of the low-risk patients; in addition, this difference was statistically significant.

### Prognostic model and prognostic value of clinicopathologic features

Clinical information (age, sex, T-staging, and N-staging) found in both the TCGA and GEO datasets were tested for their impact on prognosis. Through univariate and multivariate Cox regression analyses, we found that age, T-staging, and N-staging were associated with prognosis. Additionally, the lasso-derived prognostic model (risk score) was an independent prognostic factor for OS.

**Figure 2:**
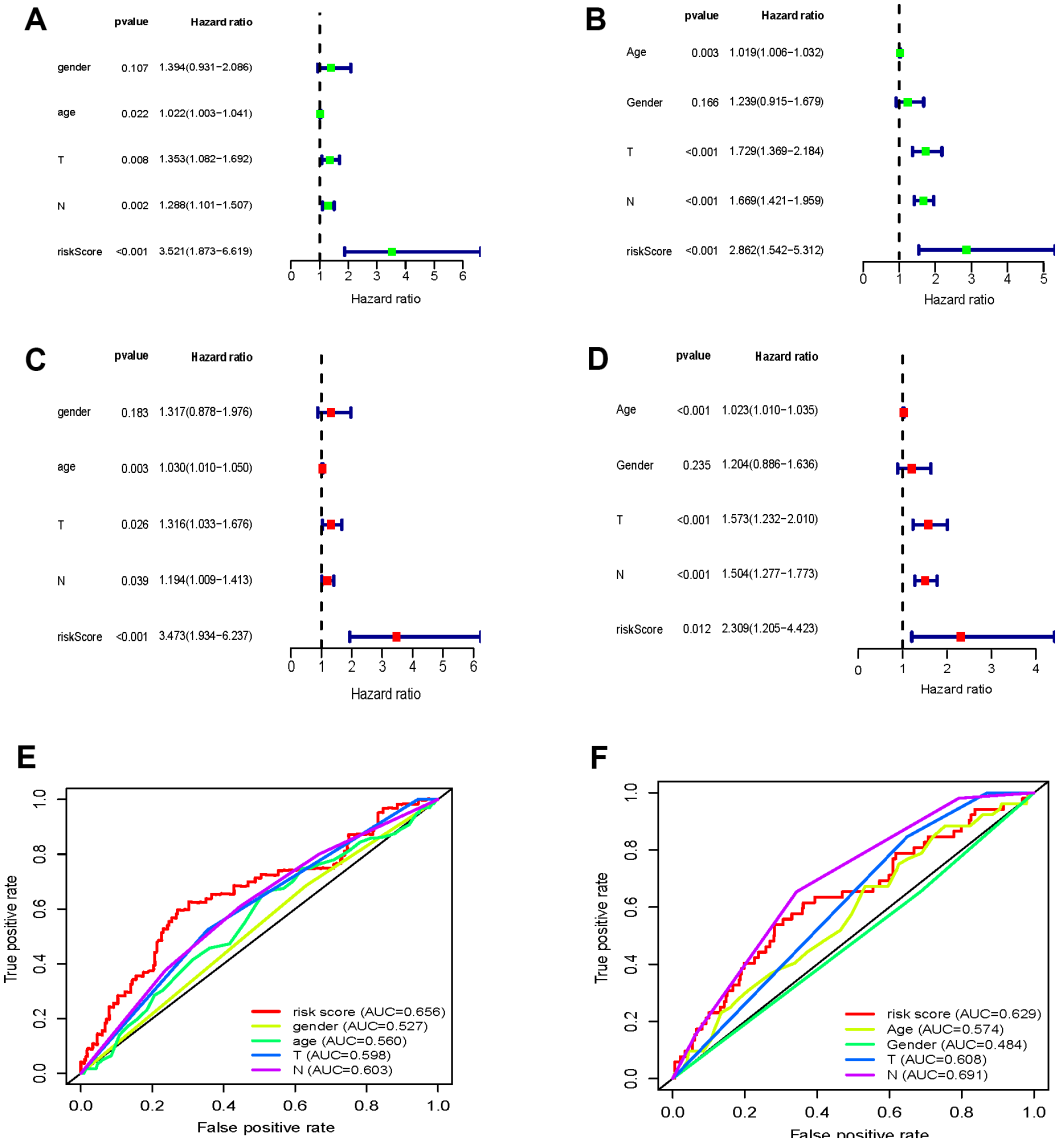
**Cell cycle-associated genes significantly correlated with survival rates of GC patients.** (**A**–**D**) Forrest plots of univariate and multivariate Cox regression analysis (**E**, **F**) OS sensitivity and specificity analysis for the risk score determined by the expression of seven genes in TCGA-STAD (**E**) and GSE84437 (**F**) based on ROC analysis.

Next, we assessed the sensitivity and specificity of the prognostic model with receiver operator characteristic (ROC) curves (Figure 2). The areas under the curve (AUCs) were 0.656 and 0.629, respectively. This indicates that the proposed model performs well, showing medium sensitivity and specificity.

### Nomogram building and validation based on the genetic model and clinical data of patients

**Figure 3:**
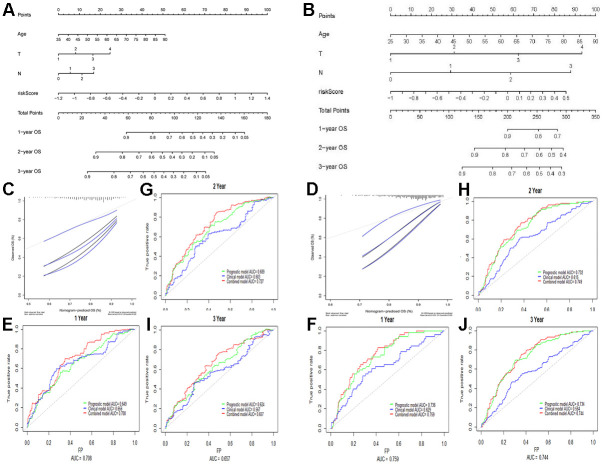
**Building and validation of the nomogram predicting the OS of GC patients in TCGA-STAD and GSE84437 datasets.** (**A**, **B**) Nomogram based on four independent prognostic factors of GC. (**C**, **D**) Calibration map for internal validation of the nomogram. (**E**–**J**) Time-dependent ROC curves of the nomogram for comparing the 1, 2, and 3 year OS of GC patients. Data in Figures (**E**, **G**, **I**) are derived from TCGA data, whereas those in Figures (**F**, **H**, **J**) are from the GSE84437 array.

A nomogram was constructed on the basis of age, T-staging, and N-staging, as well as the proposed prognostic model. As indicated by the calibration chart (Figure 3), the nomogram performed best for predicting 1 year OS. The consistency indices (C-indices) of the clinical model (involving age, T-staging, and N-staging), the prognostic model, and the nomogram model were 0.636, 0.622, and 0.677, respectively. The clinical model had a lower C-index than the nomogram model (*P* < 0.001). Combining the prognostic model and the clinical model, the AUCs of 1, 2, and 3 year OS were improved to 0.708, 0.727, and 0.657, respectively. According to our results, the nomogram model better predicted survival prognosis than the clinical model for GC.

### External immunohistochemical validation based on protein levels

**Figure 4:**
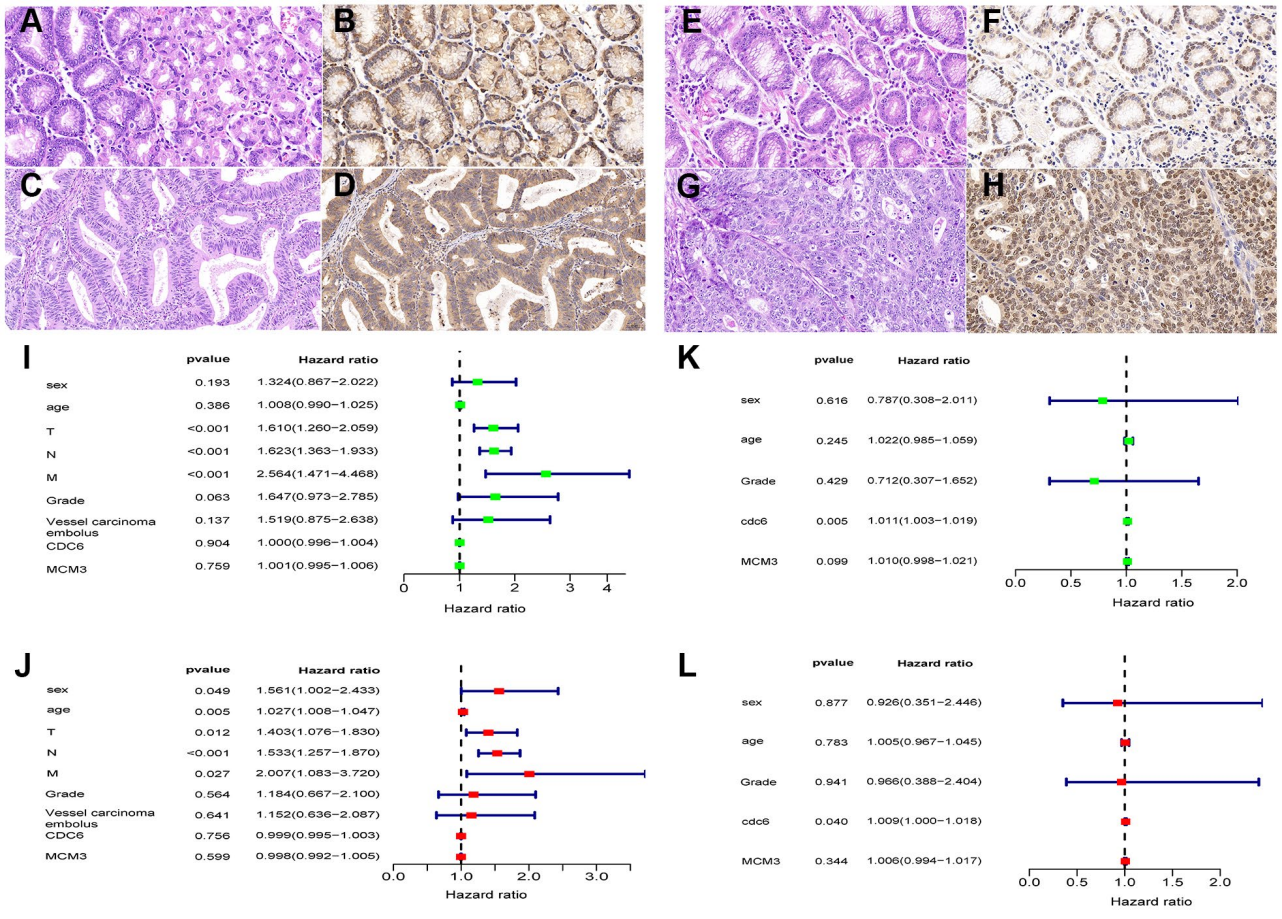
**Protein expression levels of CDC6 and MCM3 in Chinese GC patients by immunohistochemistry (IHC).** CDC6 significantly correlated with survival rates of GC patients with stratified analysis. HE-stained sections (**A**) and IHC staining of CDC6 in nontumor tissues: H-SCORE 181.1 (**B**); HE-stained sections (**C**) and IHC staining of CDC6 in tumor tissues: H-SCORE 169.8 (**D**); HE-stained sections (**E**) and IHC staining of MCM3 in nontumor tissues: H-SCORE 52.7 (**F**); HE-stained sections (**G**) and IHC staining of MCM3 in tumor tissues: H-SCORE 156.1 (**H**); Magnification: 400×. (**I**–**L**) Forrest plots of univariate and multivariate Cox regression analysis.

Protein levels of CDC6 and MCM3 from 234 Chinese patients with GC were obtained using immunohistochemistry. We found that the expression in tumor tissues of these proteins was significantly higher than that in nontumor tissues (*P* < 0.01). Among the patients, data from 232 Chinese GC patients with OS longer than 30 days (Table 1) were included in univariate and multivariate Cox regression analyses. The results showed that TNM staging were associated with prognosis (Table 1). Furthermore, CDC6 was an independent prognostic factor in GC patients with T_1-3_N_1-2_M_0_ staging and no vascular tumor thrombus as presented in the 7^th^ version of the ACJJ (*P*<0.05) (Figure 4).

### Gene set enrichment analysis

**Figure 5:**
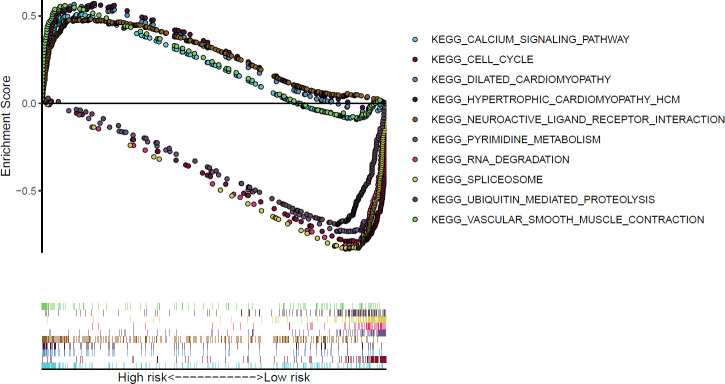
KEGG pathways in GSEA significantly enriched among differentially expressed genes from the TCGA data.

Through gene set enrichment analysis (GSEA) of the differentially expressed genes from the TCGA-STAD dataset, we found that KEGG cell cycle pathways were enriched in the low-risk group (*P*<0.001 and false discovery rate (FDR)<0.001) (Figure 5).

## DISCUSSION

In this study, we included cell cycle-related genes associated with GC to explore their potential as prognostic factors for GC. We identified seven prognosis-related cell cycle genes and established a risk prediction model. For GC patients with high- and low-risk scores, we observed different survival rates. Additionally, a nomogram created by combining clinical factors with our prognostic model performed even better in predicting the survival prognosis of GC patients, with verification in a second dataset.

As a heterogeneous disease, the development of GC is a long and multistep process. Because of the gradual accumulation of genetic mutations, carcinogenesis and anti-cancer pathway imbalances eventually give rise to GC [12]. With progress in medical technology, the morbidity and mortality of GC have both declined. However, the current prognosis of GC patients is not optimistic [13].

In cancer, abnormal activity of various cyclins leads to uncontrollable tumor cell proliferation. In the literature, it has been reported that cell cycle regulators are related to the prognosis of GC patients. For instance, in gastric adenocarcinoma tissues, downregulated expression of the protein p27 is correlated with advanced tumors [14]. Among poorly differentiated tumors, the expression levels of p27 and CCND1 were elevated and were shown to be negative prognostic factors for patient survival [15]. Overexpression of the proteins CCND1 and CCND2 is associated with the short OS of GC patients [16]. Furthermore, binding of cyclin E and cyclin-dependent kinase 2 promotes the transition of the cell cycle from stage G1 to stage S. The prognosis of GC patients positive for cyclin E is poor; combining cyclin E overexpression with p53 expression was able to differentiate patients with poor prognosis [17, 18]. Recently, bioinformatics methods were used to confirm that five G2/M checkpoint-related genes (i.e., *MARCKS*, *CCNF*, *MAPK14*, *INSENP*, and *CHAF1A*) were related to the OS of GC patients [19]. Nevertheless, these studies have not established a prognostic model for GC prediction based on the genetic characterization of genes associated with the cell cycle.

In recent years, mRNA expression has been investigated on the basis of whole-genome sequencing data [20]. Using these data, studies have investigated *TP53* mutations and mutations in autophagy-related genes to construct prognostic models, showing that these models can successfully predict GC prognosis [10, 11]. In this study, CDC6 and MCM3 showed the greatest differences and were most strongly correlated with prognosis in the TCGA dataset. MCM3 falls into a family of six highly conserved minichromosomal maintenance proteins, MCM2–MCM7. These proteins are critical for ensuring that eucaryon DNA replication takes place only once in each cell cycle. Additionally, MCM3 also serves as a helicase to facilitate replication extension. At an advanced stage of M1, CDT1 and CDC6 recruit iso-hexamer MCM2–MCM7 complexes and load them onto an origin of replication, producing prereplication complexes [21]. Additionally, CDC6 is an important cell cycle regulator [22]. By assembling prereplication complexes, it plays an essential role in maintaining chromosome integrity [23, 24]. Moreover, CDC6 is also closely related to tumorigenesis. Besides playing a role in cancer-related pathways by regulating the expression of certain oncogenes and cancer suppressor genes such as *KRAS* [25], CDC6 promotes cancer progression as it is positively regulated by associated noncoding RNAs (e.g., *lncRNA-CDC6*) [26]. Furthermore, the expression of CDC6 is upregulated in multiple tumor types [27, 28]; CDC6 serves as a prognostic biomarker of breast carcinoma [29], colorectal cancer [30], pancreatic cancer [31], and other cancers. CDC6 knockdown has been shown to inhibit the growth and invasion of GC cells. Transfection of pS-CDC6 knocked down CDC6 expression in the GC cell lines BGC823 and SGC7901. MTT assay showed that transfection of pS-CDC6 markedly inhibited cell proliferation in BGC823 and SGC7901 cells. Transwell assay revealed that pS-CDC6 transfection inhibited the invasive capacities of BGC823 and SGC7901 cells. This likely be due to CDC6 knockdown promoting the apoptosis of these cells [32]. Among 232 Chinese GC patients, validation through immunohistochemical methods showed that the protein levels of CDC6 and MCM3 in tumor and nontumor tissues had significantly different histochemistry scores (H-scores; see Methods). Additionally, we showed that CDC6 is an independent prognostic factor for GC with stratified analysis. Finally, via KEGG enrichment analysis, we showed that the gene expression in the low-risk group was related to cell cycle pathways, and patients in this group may benefit more from cell cycle therapies.

In summary, cell cycle genes play an important role in cancer progression. This study shows the value of cell cycle genes as prognostic biomarkers for GC. However, as a retrospective study, certain limitations are inevitable. To understand the specific mechanism and biological functions of cell cycle genes on prognosis, further investigation is needed.

## Conclusions

We constructed a prognostic model involving seven cell cycle genes based on GC data from TCGA and GEO. Combining the proposed prognostic model with a nomogram of clinical pathology, a higher survival prognosis prediction ability can be achieved. According to our findings, the proposed prognostic model may promote individualized diagnosis and treatment of GC patients and thus further improve the prognosis of these patients.

## MATERIALS AND METHODS

### Gene expression data

On the TCGA website, we downloaded RNA sequencing and clinical data involving 375 GC tissues and 32 nontumor tissues from the STAD dataset. Subsequently, 125 cell cycle-related genes were obtained from the KEGG database, and GSE84437 data were downloaded from the GEO database. These datasets were used for further analysis after they were corrected by the “batchType” correction model in the SVA4.04R software package.

### Differentially expressed cell cycle genes in GC

Using the “limma” package in R, 375 tumor samples and 32 normal tissue samples from TCGA were subjected to differential expression analysis for cell cycle genes. Differentially expressed genes were selected with an FDR of <0.05 and log fold-change of >0.5.

### Prognostic gene model

Univariate Cox proportional hazards regression analysis was conducted to select cell cycle genes significantly correlated with the OS of individuals included in the TCGA-STAD dataset. According to the hazard ratio of each gene and *P*-values obtained using the Wald test, *P*<0.05 was adopted as the threshold to select genes that were significantly correlated with the survival of GC patients. Based on the “glmnet” package in R [33, 34], a multivariate model of cell cycle genes was constructed via lasso regression. In the lasso regression model, genes with nonzero coefficients were selected to calculate risk scores by the following equation: risk score=∑^n^_j=1_Coefj*Xj, where Coef j stands for the coefficient and Xj for the relative expression level of each cell cycle gene [35]. All TCGA patients were divided into two groups (i.e., a high-risk group and a low-risk group) depending on the median risk score. Similar to the TCGA dataset, the same formula was used to determine the risk scores in the GEO dataset. We then calculated Kaplan–Meyer survival curves and used the log-rank test to clarify the statistical significance of the difference in OS between the two groups. Clinical information (e.g., age, sex, T-staging, and N-staging) was then extracted from the TCGA-STAD and GSE84437 datasets. These factors were tested for their impact on prognosis with univariate and multivariate Cox regression analyses. Any factor with a *P*<0.05 was deemed statistically significant. Moreover, ROC curves were also calculated to verify the predictive value of the model.

### Nomogram-based prediction model

Survival data from the patients were combined with their age, T-staging, N-staging, and risk scores to build a nomogram using the “rms” package in R. Subsequently, calibration curves were calculated to evaluate the consistency between the actual and predicted survival rates. Furthermore, a C-index ranging from 0.5 to 1.0 was calculated to assess the performance of the prognosis prediction model. The AUC was used to evaluate the sensitivity and specificity of the model.

### Immunohistochemical evaluation of protein expression levels of cell cycle genes

Two hundred fifty tumor tissues and 144 nontumor tissues were derived from 250 Chinese patients affected by GC. From the TCGA database, CDC6 and MCM3, which were most closely correlated with prognosis, were selected for validation based on their protein levels. The H-score is used to evaluate immunohistochemical conditions. In detail, the number of positive cells on each slide and their staining intensity are converted into values as a semiquantitative measure of the amount of histological staining. H-Score (H-SCORE=∑pi × i)=(percentage of weak intensity cells ×1)+(percentage of moderate intensity cells ×2)+(percentage of strong intensity cells ×3), where pi represents the proportion of positive cells and i is the staining intensity. The H-score has a range of 0–300. The greater the value is, the greater the staining intensity of positive cells will be [36, 37].

### Gene set enrichment analyses

Regarding the differentially expressed genes in the TCGA-STAD array between the high- and low-risk groups, GSEA was used to identify enriched pathways [38, 39]. *P*<0.05 and FDR<0.25 were considered statistically significant.

### Statistical analysis

All statistical analyses were conducted in R software (Version 4.0.4; https://www.R-project.org). The correlation between the risk scores and clinical features was investigated using the χ^2^ test. Besides drawing Kaplan–Meier curves, a log-rank test was conducted to test intergroup differences in OS. Univariate and multivariate Cox proportional risk regression analyses were conducted to determine the association between the risk score and OS. The AUC was calculated as a measure of the prediction accuracy of the prognostic models. *P*<0.05 was used to signify statistically significant results.

## References

[1] Choi RS, Lai WYX, Lee LTC, Wong WLC, Pei XM, Tsang HF, Leung JJ, Cho WCS, Chu MKM, Wong EYL, Wong SCC. Current and future molecular diagnostics of gastric cancer. Expert Rev Mol Diagn. 2019; 19:863–74. 10.1080/14737159.2019.1660645 31448971

[2] Machlowska J, Maciejewski R, Sitarz R. The Pattern of Signatures in Gastric Cancer Prognosis. Int J Mol Sci. 2018; 19:1658. 10.3390/ijms19061658 29867026 PMC6032410

[3] Min A, Kim JE, Kim YJ, Lim JM, Kim S, Kim JW, Lee KH, Kim TY, Oh DY, Bang YJ, Im SA. Cyclin E overexpression confers resistance to the CDK4/6 specific inhibitor palbociclib in gastric cancer cells. Cancer Lett. 2018; 430:123–32. 10.1016/j.canlet.2018.04.037 29729292

[4] Torre LA, Bray F, Siegel RL, Ferlay J, Lortet-Tieulent J, Jemal A. Global cancer statistics, 2012. CA Cancer J Clin. 2015; 65:87–108. 10.3322/caac.21262 25651787

[5] Camargo MC, Kim WH, Chiaravalli AM, Kim KM, Corvalan AH, Matsuo K, Yu J, Sung JJ, Herrera-Goepfert R, Meneses-Gonzalez F, Kijima Y, Natsugoe S, Liao LM, et al. Improved survival of gastric cancer with tumour Epstein-Barr virus positivity: an international pooled analysis. Gut. 2014; 63:236–43. 10.1136/gutjnl-2013-304531 23580779 PMC4384434

[6] Szczepanik A, Sierzega M, Drabik G, Pituch-Noworolska A, Kołodziejczyk P, Zembala M. CD44^+^ cytokeratin-positive tumor cells in blood and bone marrow are associated with poor prognosis of patients with gastric cancer. Gastric Cancer. 2019; 22:264–72. 10.1007/s10120-018-0858-2 30056567 PMC6394724

[7] Cho SJ, Kook MC, Lee JH, Shin JY, Park J, Bae YK, Choi IJ, Ryu KW, Kim YW. Peroxisome proliferator-activated receptor γ upregulates galectin-9 and predicts prognosis in intestinal-type gastric cancer. Int J Cancer. 2015; 136:810–20. 10.1002/ijc.29056 24976296

[8] Lin C, Liu H, Zhang H, He H, Li H, Shen Z, Qin J, Qin X, Xu J, Sun Y. Interleukin-13 receptor α2 is associated with poor prognosis in patients with gastric cancer after gastrectomy. Oncotarget. 2016; 7:49281–8. 10.18632/oncotarget.10297 27351230 PMC5226507

[9] He Q, Li G, Wang X, Wang S, Hu J, Yang L, He Y, Pan Y, Yu D, Wu Y. A Decrease of Histone Deacetylase 6 Expression Caused by Helicobacter Pylori Infection is Associated with Oncogenic Transformation in Gastric Cancer. Cell Physiol Biochem. 2017; 42:1326–35. 10.1159/000478961 28700998

[10] Nie K, Zheng Z, Wen Y, Shi L, Xu S, Wang X, Zhou Y, Fu B, Li X, Deng Z, Pan J, Jiang X, Jiang K, et al. Construction and validation of a TP53-associated immune prognostic model for gastric cancer. Genomics. 2020; 112:4788–95. 10.1016/j.ygeno.2020.08.026 32858135

[11] Chen L, Ma G, Wang P, Dong Y, Liu Y, Zhao Z, Guo J, Liang H, Yang L, Deng J. Establishment and verification of prognostic model for gastric cancer based on autophagy-related genes. Am J Cancer Res. 2021; 11:1335–46. 33948361 PMC8085875

[12] Molina-Castro S, Pereira-Marques J, Figueiredo C, Machado JC, Varon C. Gastric cancer: Basic aspects. Helicobacter. 2017 (Suppl 1); 22:e12412. 10.1111/hel.12412 28891129

[13] Parkin DM. The global health burden of infection-associated cancers in the year 2002. Int J Cancer. 2006; 118:3030–44. 10.1002/ijc.21731 16404738

[14] Nitti D, Belluco C, Mammano E, Marchet A, Ambrosi A, Mencarelli R, Segato P, Lise M. Low level of p27(Kip1) protein expression in gastric adenocarcinoma is associated with disease progression and poor outcome. J Surg Oncol. 2002; 81:167–75. 10.1002/jso.10172 12451619

[15] Shan YS, Hsu HP, Lai MD, Hung YH, Wang CY, Yen MC, Chen YL. Cyclin D1 overexpression correlates with poor tumor differentiation and prognosis in gastric cancer. Oncol Lett. 2017; 14:4517–26. 10.3892/ol.2017.6736 28943959 PMC5594254

[16] Ding ZY, Li R, Zhang QJ, Wang Y, Jiang Y, Meng QY, Xi QL, Wu GH. Prognostic role of cyclin D2/D3 in multiple human malignant neoplasms: A systematic review and meta-analysis. Cancer Med. 2019; 8:2717–29. 10.1002/cam4.2152 30950241 PMC6558476

[17] Alsina M, Landolfi S, Aura C, Caci K, Jimenez J, Prudkin L, Castro S, Moreno D, Navalpotro B, Tabernero J, Scaltriti M. Cyclin E amplification/overexpression is associated with poor prognosis in gastric cancer. Ann Oncol. 2015; 26:438–9. 10.1093/annonc/mdu535 25403579

[18] Bani-Hani KE, Almasri NM, Khader YS, Sheyab FM, Karam HN. Combined evaluation of expressions of cyclin E and p53 proteins as prognostic factors for patients with gastric cancer. Clin Cancer Res. 2005; 11:1447–53. 10.1158/1078-0432.CCR-04-1730 15746045

[19] Zhao L, Jiang L, He L, Wei Q, Bi J, Wang Y, Yu L, He M, Zhao L, Wei M. Identification of a novel cell cycle-related gene signature predicting survival in patients with gastric cancer. J Cell Physiol. 2019; 234:6350–60. 10.1002/jcp.27365 30238991

[20] Dai J, Li ZX, Zhang Y, Ma JL, Zhou T, You WC, Li WQ, Pan KF. Whole Genome Messenger RNA Profiling Identifies a Novel Signature to Predict Gastric Cancer Survival. Clin Transl Gastroenterol. 2019; 10:e00004. 10.14309/ctg.0000000000000004 30702489 PMC6369880

[21] Hyrien O, Marheineke K, Goldar A. Paradoxes of eukaryotic DNA replication: MCM proteins and the random completion problem. Bioessays. 2003; 25:116–25. 10.1002/bies.10208 12539237

[22] Kim GS, Lee I, Kim JH, Hwang DS. The Replication Protein Cdc6 Suppresses Centrosome Over-Duplication in a Manner Independent of Its ATPase Activity. Mol Cells. 2017; 40:925–34. 10.14348/molcells.2017.0191 29237113 PMC5750711

[23] Borlado LR, Méndez J. CDC6: from DNA replication to cell cycle checkpoints and oncogenesis. Carcinogenesis. 2008; 29:237–43. 10.1093/carcin/bgm268 18048387

[24] Teixeira LK, Reed SI. Cdc6: Skin in the carcinogenesis game. Cell Cycle. 2016; 15:313. 10.1080/15384101.2015.1131528 26694635 PMC4943738

[25] Rossi E, Klersy C, Manca R, Zuffardi O, Solcia E. Correlation between genomic alterations assessed by array comparative genomic hybridization, prognostically informative histologic subtype, stage, and patient survival in gastric cancer. Hum Pathol. 2011; 42:1937–45. 10.1016/j.humpath.2011.02.016 21676433

[26] Kong X, Duan Y, Sang Y, Li Y, Zhang H, Liang Y, Liu Y, Zhang N, Yang Q. LncRNA-CDC6 promotes breast cancer progression and function as ceRNA to target CDC6 by sponging microRNA-215. J Cell Physiol. 2019; 234:9105–17. 10.1002/jcp.27587 30362551

[27] Kim YH, Byun YJ, Kim WT, Jeong P, Yan C, Kang HW, Kim YJ, Lee SC, Moon SK, Choi YH, Yun SJ, Kim WJ. CDC6 mRNA Expression Is Associated with the Aggressiveness of Prostate Cancer. J Korean Med Sci. 2018; 33:e303. 10.3346/jkms.2018.33.e303 30450027 PMC6236078

[28] Deng Y, Jiang L, Wang Y, Xi Q, Zhong J, Liu J, Yang S, Liu R, Wang J, Huang M, Tang C, Su M. High expression of CDC6 is associated with accelerated cell proliferation and poor prognosis of epithelial ovarian cancer. Pathol Res Pract. 2016; 212:239–46. 10.1016/j.prp.2015.09.014 26920249

[29] Mahadevappa R, Neves H, Yuen SM, Bai Y, McCrudden CM, Yuen HF, Wen Q, Zhang SD, Kwok HF. The prognostic significance of Cdc6 and Cdt1 in breast cancer. Sci Rep. 2017; 7:985. 10.1038/s41598-017-00998-9 28428557 PMC5430515

[30] Hu Y, Wang L, Li Z, Wan Z, Shao M, Wu S, Wang G. Potential Prognostic and Diagnostic Values of CDC6, CDC45, ORC6 and SNHG7 in Colorectal Cancer. Onco Targets Ther. 2019; 12:11609–21. 10.2147/OTT.S231941 32021241 PMC6942537

[31] Yan X, Wan H, Hao X, Lan T, Li W, Xu L, Yuan K, Wu H. Importance of gene expression signatures in pancreatic cancer prognosis and the establishment of a prediction model. Cancer Manag Res. 2018; 11:273–83. 10.2147/CMAR.S185205 30643453 PMC6312063

[32] Zhao B, Zhang J, Chen X, Xu H, Huang B. Mir-26b inhibits growth and resistance to paclitaxel chemotherapy by silencing the CDC6 gene in gastric cancer. Arch Med Sci. 2019; 15:498–503. 10.5114/aoms.2018.73315 30899303 PMC6425209

[33] Friedman J, Hastie T, Tibshirani R. Regularization Paths for Generalized Linear Models via Coordinate Descent. J Stat Softw. 2010; 33:1–22. 20808728 PMC2929880

[34] Sauerbrei W, Royston P, Binder H. Selection of important variables and determination of functional form for continuous predictors in multivariable model building. Stat Med. 2007; 26:5512–28. 10.1002/sim.3148 18058845

[35] Kidd AC, McGettrick M, Tsim S, Halligan DL, Bylesjo M, Blyth KG. Survival prediction in mesothelioma using a scalable Lasso regression model: instructions for use and initial performance using clinical predictors. BMJ Open Respir Res. 2018; 5:e000240. 10.1136/bmjresp-2017-000240 29468073 PMC5812388

[36] Maclean A, Bunni E, Makrydima S, Withington A, Kamal AM, Valentijn AJ, Hapangama DK. Fallopian tube epithelial cells express androgen receptor and have a distinct hormonal responsiveness when compared with endometrial epithelium. Hum Reprod. 2020; 35:2097–106. 10.1093/humrep/deaa177 32876325

[37] Dogan S, Vasudevaraja V, Xu B, Serrano J, Ptashkin RN, Jung HJ, Chiang S, Jungbluth AA, Cohen MA, Ganly I, Berger MF, Momeni Boroujeni A, Ghossein RA, et al. DNA methylation-based classification of sinonasal undifferentiated carcinoma. Mod Pathol. 2019; 32:1447–59. 10.1038/s41379-019-0285-x 31186531 PMC7391258

[38] Mootha VK, Lindgren CM, Eriksson KF, Subramanian A, Sihag S, Lehar J, Puigserver P, Carlsson E, Ridderstråle M, Laurila E, Houstis N, Daly MJ, Patterson N, et al. PGC-1alpha-responsive genes involved in oxidative phosphorylation are coordinately downregulated in human diabetes. Nat Genet. 2003; 34:267–73. 10.1038/ng1180 12808457

[39] Subramanian A, Tamayo P, Mootha VK, Mukherjee S, Ebert BL, Gillette MA, Paulovich A, Pomeroy SL, Golub TR, Lander ES, Mesirov JP. Gene set enrichment analysis: a knowledge-based approach for interpreting genome-wide expression profiles. Proc Natl Acad Sci USA. 2005; 102:15545–50. 10.1073/pnas.0506580102 16199517 PMC1239896

